# Automated extraction and semantic analysis of mutation impacts from the biomedical literature

**DOI:** 10.1186/1471-2164-13-S4-S10

**Published:** 2012-06-18

**Authors:** Nona Naderi, René Witte

**Affiliations:** 1 Semantic Software Lab, Department of Computer Science and Software Engineering, Concordia University, Montréal, Québec, H2K 3V4, Canada

## Abstract

**Background:**

Mutations as sources of evolution have long been the focus of attention in the biomedical literature. Accessing the mutational information and their impacts on protein properties facilitates research in various domains, such as enzymology and pharmacology. However, manually curating the rich and fast growing repository of biomedical literature is expensive and time-consuming. As a solution, text mining approaches have increasingly been deployed in the biomedical domain. While the detection of single-point mutations is well covered by existing systems, challenges still exist in grounding impacts to their respective mutations and recognizing the affected protein properties, in particular kinetic and stability properties together with physical quantities.

**Results:**

We present an ontology model for mutation impacts, together with a comprehensive text mining system for extracting and analysing mutation impact information from full-text articles. Organisms, as sources of proteins, are extracted to help disambiguation of genes and proteins. Our system then detects mutation series to correctly ground detected impacts using novel heuristics. It also extracts the affected protein properties, in particular kinetic and stability properties, as well as the magnitude of the effects and validates these relations against the domain ontology. The output of our system can be provided in various formats, in particular by populating an OWL-DL ontology, which can then be queried to provide structured information. The performance of the system is evaluated on our manually annotated corpora. In the impact detection task, our system achieves a precision of 70.4%-71.1%, a recall of 71.3%-71.5%, and grounds the detected impacts with an accuracy of 76.5%-77%. The developed system, including resources, evaluation data and end-user and developer documentation is freely available under an open source license at http://www.semanticsoftware.info/open-mutation-miner.

**Conclusion:**

We present Open Mutation Miner (OMM), the first comprehensive, fully open-source approach to automatically extract impacts and related relevant information from the biomedical literature. We assessed the performance of our work on manually annotated corpora and the results show the reliability of our approach. The representation of the extracted information into a structured format facilitates knowledge management and aids in database curation and correction. Furthermore, access to the analysis results is provided through multiple interfaces, including web services for automated data integration and desktop-based solutions for end user interactions.

## Background

Vast amounts of research is dedicated to the identification of mutations and their impacts. Biologists usually make inferences about functions of novel sequences by comparing them to the functions of known sequences [1]. Many mutagenesis experiments are performed to improve the properties of proteins, particularly enzymes. Additionally detection of disease causal mutations attracted a lot of attention. The result of all these efforts lies in publications, particularly in textual format. Consequently, locating and retrieving this information is a very cumbersome task. Some databases try to manually curate such information and provide it in publicly accessible form. However, even for an expert curator, extracting this information manually is laborious. Hence, database curators now increasingly reach for text-mining procedures.

Large-scale attempts resulted in high levels of performance in the realization of the automatic extraction of mutations [2-10]. Yet, finding their *impacts*, *affected protein properties* and *magnitudes of effects* remains challenging.

MEMA [2] uses regular expressions to extract mutations and mutation-gene pairs. It focuses on the co-occurrence of mutations and genes within a sentence and proximity parameters within an abstract. The performance of the system is evaluated on a set of 100 abstracts. The reported recall and precision for the mutation detection task are >67% and >96%, respectively.

MuteXt [3] searches for mutation data using a pattern matching approach and further validates the extracted point mutations using two plausibility filters: A sequence filter and distance filter. The performance of the system is evaluated on two corpora. Their algorithm detects 49.3%-64.5% of point mutations with a specificity of 85.8%-87.9%.

Mutation GraB [4] takes a dictionary-based approach to identify protein and gene names while extracting point mutation terms using regular expressions, utilizing graph bigrams to disambiguate the extracted protein point mutations. The authors evaluate the effectiveness of their approach on the articles describing three protein families, namely, *tyrosine protein kinases*, *GPCRs* and *transmembrane ion.*

MuGex [5] uses 12 regular expressions to detect mutations and statistical techniques to disambiguate between protein mutations and nucleotide mutations or cell lines. Gene-mutation pairs are detected through proximity measures.

The MutationFinder system [6] extends MuteXt [3]’s rules to extract and normalize point mutations.

In recent work [7], the authors present a strategy to integrate information about phenotypic effect of SNPs from UniProtKB and pathways from Reactom and BioPAX for visualization in Cytoscape.

Yip et al. [8] uses 4 regular expressions to extract and retrieve single amino acid poly morphisms (SAPs). The system is assessed on a Swiss-Prot corpus with 9820 PubMed references. Additionally, each pattern is evaluated separately.

The mSTRAP (Mutation extraction and STRucture Annotation Pipeline) system [9] was developed with the aim of annotating mutations and representing them as instances of an ontology. They further use mSTRAPviz to read the populated ontology and visualize the annotations on protein structures.

EnzyMiner [10] tries to categorize PubMed abstracts based on the impact of a protein level mutation on the stability and activity of a given enzyme. Using different classification algorithms, EnzyMiner is able to narrow down search results; however, detailed information about the direction of the impacts, association of impacts to mutations and the kind of change in stability or functionality is not provided. Although EnzyMiner targets mutation impact information, it differs significantly from our approach, as we are concerned with sentence-level detection and semantic analysis of mutation impacts, not document classification.

In [11], the authors introduced the first rule-based approach to extract mutation impacts on protein properties while categorizing the directionality of the impacts and grounding the impacts to the mutations. The extracted information was populated to a domain ontology for further querying through a web service. While in the aforementioned work, molecular properties and the Michaelis constant (*K_m_*), the rate constant (*K_cat_*) and the compound variable (*K_cat_* / *K_m_*) are considered, the other protein properties, such as the remaining kinetic constants and protein stability, are ignored. On the corpus of 13 documents on haloalkane dehalogenase, the authors report a recall of 34% and a precision of 86% for the mutation-impact relation extraction task.

A recent work on the extraction of kinetic information and associated information, namely, enzyme names, EC numbers and localization is presented in [12]. The proposed rule- and dictionary-based approach in this system is applied to PubMed abstracts and the results are provided in *KID*, *the KInetic Database *[13].

KiPar [14], an information retrieval system, focuses on kinetic modeling of metabolic pathways using a rule-based approach.

However, all the existing approaches are unable to extract the protein properties affected by mutations. In this paper, we present a rule-based approach to extract mutation series, modified protein properties and magnitudes of effects [15,16]. In our system, the relation between the magnitudes of effects and the protein properties are detected and validated against the domain ontology. To provide for effective querying and analysis, we populate a domain ontology with the extracted information. Table 1 summarizes the scope of our Open Mutation Miner (OMM) system, compared to existing approaches. Further details on these tasks are provided in the following section.

**Table 1:** Literature mining approaches for mutations and impacts

	**MEMA**[**2**]	**MuteXt**[**3**]	**Mutation GraB**[**4**]	**MuGex**[**5**]	**mSTRAP**[**9**]	**Mutation Miner**[**20**]	**MutationFinder**[**6**]	**Yip et al.**[**8**]	**Mehren et al.**[**7**]	**Laurila et al.**[**11**]	OMM
**Mutation Tagging**	√	√	√	√	√	√	√	√		√	√
**Mutation Series Tagging**						(√)					√
**Mutation-Protein Grounding**	√	√	√		√	√			√		

**Impact Tagging**										√	√
**Impact-Mutation Grounding**										√	√
**Protein Property Tagging**										(√)	√
**Physical Quantity Tagging**											√
**Impact-Protein Property Grounding**											√
**Protein Property-Physical Quantity Grounding**											√

**Visualization**					√	√			√		
**Ontology Export**					√					(√)	√
**Web Service Access**										(√)	√

## Methods

In order to comprehensively extract mutation impacts, the detection of several named entities and their relations, in particular *mutations* and *protein properties*, is required. As an example, consider the following text segment (formatting used: ***bold face***: Mutation; *underlined*: Impact expression; underlined non-italics: Protein property; ***underlined bold***: Physical quantity) [17]: *“Several single mutants* (*Q15K*, *Q15R*, *W37K*, *and W37R*), *double mutants* (*Q15K-W37K*, *Q15K-W37R*, *Q15R-W37K*, *and Q15R-W37R*), *and triple mutants* (*Q15K-D36A-W37R and Q15K-D36S-W37R*) *were prepared and expressed as glutathione S-transferase* (*GST*) *fusion proteins in Escherichia coli and purified by GSH-agarose affinity chromatography. Mutant ****Q15K-W37R**** and mutant ****Q15R-W37R**** showed comparable activity for NAD and NADP with an increase in *activity* nearly ****3fold**** over that of the wild type.“*

In this example, we need to extract *increase* as an impact that is caused “comparably” by two mutation pairs, *Q15K-W37R* and *Q15R-W37R.* In other words, the two aforementioned mutations have the same impact on the *activity* of an enzyme, *glutathione S-transferase* (*GST*) that is residing in the host organism, *Escherichia coli.* We are also interested to know that *activity* as a kinetic property of the mutant enzyme is measured *3fold* higher than the activity of the wild-type enzyme. Note that other entities, such as single mutations (*Q15K*, *Q15R*, *W37K*, and *W37R*), exist in the text segment, but here we are only interested in the entities that are related to the identified impact. The result of the system should be a set of detected entities, correctly normalized and grounded, and linked with each other.

**Figure 1:**
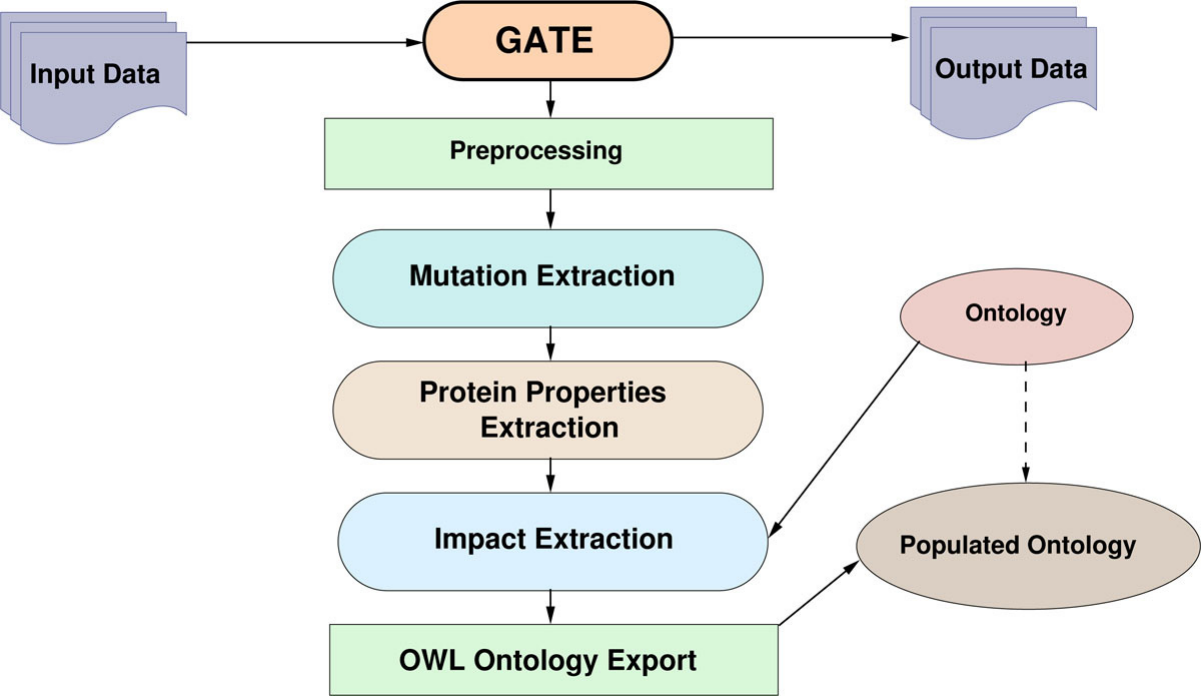
**Open Mutation Miner (OMM) System Overview** Input documents are processed through a text mining pipeline implemented in GATE, which (1) performs preprocessing; (2) detects mutation mentions; (3) detects protein properties; and (4) detects impact mentions and links the detected entities. Results can be exported in various formats, in particular by populating the OMM ontology.

After detecting organism mentions, which is handled by a separate module, the OrganismTagger [18], the first step of impact analysis is to detect impact mentions. However, extracting only impacts is not sufficient; we want to know which mutation *caused* the impact. Hence, the system needs to ground the detected impacts to mutations. Additionally, mutations can appear in the form of mutation series (see the above example). Thus, the system must also be able to identify these complex mutation expressions. Finding out which protein properties were affected by the mutations and to what extent is necessary to identify advantageous mutations. Towards this end, we export the analysis results into an ontology (so-called ontology population [19]) for further applications, including queries and summarization. An overview of our system is presented in Figure 1. In what follows, we will provide a detailed description of each task.

### Impact ontology

**Figure 2:**
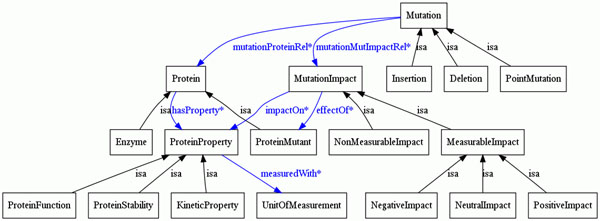
**OMM impact ontology** Visualization of the main concepts in the OMM Impact Ontology, which formally describes the domain of mutation impact analysis using the Web Ontology Language (OWL).

Our Impact Ontology is an extension to the ontology described in [20], conceptualizing impacts and the mutations associated with them (Figure 2). The use of the impact ontology facilitates advanced queries and impact extraction. The ontology contains information about several concepts: Text elements, biological entities and entity relations, e.g., *Sentence*, *Mutationlmpact* and *measuredWith*, respectively. We extended the ontology with new classes, such as *MichaelisMentenConstant*, *SpecificActivity*, *and MaximalVelocity.* Our ontology has a rich set of relationships between the concepts. Main concepts modeling impacts on a semantic level are:

**Mutation:** An alteration or a change to a gene and developing a different offspring.

**UnitOfMeasurement:** A standard for measuring the physical quantity.

**Mutationlmpact:** The expansion of an impact can be presented as a bifurcating tree: each bifurcating node represents a mutation effect on protein properties, whether the impact is measurable or not.

**ProteinProperty:** A class for protein properties, which subsumes *kinetic properties*, *protein function*, and *protein stability.*

Information about the effect of mutations on proteins can be modeled at different granularity levels. For example, the effect can be on the structure, which consequently can affect various properties of the proteins. For a finer level of granularity, we represent all these relations. The relations between these entities, expressed as OWL object properties, are listed in Table 2.

**Table 2:** Mutation impact concepts in the Open Mutation Miner ontology

Object Property	Domain	Range	Description
hasProperty	Protein	ProteinProperty	Which protein the protein property belongs to
impactOn	Mutlmpact	ProteinProperty	Identifies the protein property affected by a mutation
measuredWith	ProteinProperty	UnitOfMeasurement	Holds between protein property and the corresponding unit of measurement
mutationMutlmpactRel	Mutation	Mutlmpact	Associates an impact with a mutation

**Datatype Property**	**Domain**	**Range**	**Description**

physicalQuantity	UnitOfMeasurement	value	Identifies the magnitude of a mutation effect on the protein property

Each protein property is measured with specific units of measurement, for example *Michaelis Menten Constant* is measured with units such as *per second*, *per minute*, etc. However, in interpreting the mutation impacts, not only are these units of measurement utilized, but also ratio measurements can be used. For example, the measured values of the affected protein property are compared with the measured values of the wild type or other mutated protein properties, and specified by *percent*, *fold* or *orders of magnitude.* We decided to establish some restrictions on the units of measurement with which each protein property is measured, as well as the ratio measurement units. These restrictions are encoded in the ontology based on global standards (SI [21]), where protein properties are measured by specific units of measurements. These constraints are encoded as possible value fillers for the *measuredWith* slot for a specific protein property. For instance, *Km* can be measured with *fold*, *per second* and *per minute*, etc. We also defined a datatype property for protein properties, called *physicalQuantity*, referring to the value and the unit of measurement found in the text.

### Mutation extraction component

Single point mutations can be expressed in single-letter standard format or through more complex representations. We integrated one external mutation detection system and also developed our own approach.

#### MutationTagger

Our MutationTagger, based on previous work [20], extracts single point mutations using grammar rules and normalizes them to their single-letter format.

However, mutational mentions in the form of natural language, such as “Met for Val substitution found at position 270” are currently ignored by our system.

#### MutationFinder

The MutationFinder system [6] accomplishes the task of single mutation detection and normalization by using regular expressions. MutationFinder also tries to identify mutational changes expressed in natural language. However, it still fails at extracting all mentions.

### Mutation series

In the simplest case, impacts are results of single mutations. However, impacts are occasionally caused by mutation series. For example, in our corpus of 40 full-text documents, 6% of the mutation mentions are mutation series.

During the manual inspection of several hundred documents containing mutation series, it became obvious that these mutation series (complex mutation expressions) may have different appearances and representations. They can be described using special symbols, such as “/” and “:”, or keywords, such as *double mutants*, *triple mutants*, *etc.* Table 3 summarizes these different forms.

**Table 3:** Mutation series examples

notation	Mutation Series
**:**	The double mutant **Thr48Ser: Trp93Ala** and the triple mutant **Thr48Ser:Trp57Met : Trp93Ala** ...

**/**	For G223D, H225N, **G223D/T224I, T224I/H225N**, and **E156/173D** mutants, ...

**-**	Kinetic studies of the mutant **A14S-Q15K-D36S- W37R** indicated that the apparent Km . . .

**double**	**a double mutant (N190V and W191S)** and **triple mutant (Q137M, L143F and H146L)** resulted . . .

**triple**	**the triple mutant, R501A,R451A,K439A**, which eliminates all of . . .

**quadruple**	**E130D/S325T/S477G/Q481K quadruple mutations** in wild-type E. coli XL1-Blue.

**quintuple**	…**The quintuple mutant V26T R47F A74G F87V L188K** of P450BM-3(P450BM-3 QM) converts …

**+**	The reaction of the **D179W+R258E+R272D** variant of CiP with . . .

Therefore, mutations connected with these special characters or preceded by the keywords are considered as mutation series and detected through regular expressions. To ensure that these detected mutation series have one identical internal representation, we further normalize them to the format, where all the mutations in a series are separated by the notation “/”.

### Protein properties extraction component

Mutations can alter the structure of proteins that subsequently results in affecting their functions, either by gaining a function or losing one. Mutations may also affect the stability of proteins, where the ratio of the unfolded protein increases or decreases compared to the folded protein. Mutagenesis experiments are constantly performed to identify the importance of protein residues, either to find the source of a disease or a cure to one. Furthermore, studies are done to improve enzyme functions.

In our system, protein properties are expressed in RDF format [22] and detected through gazetteering.

The extracted information can then be correlated to impacts in subsequent processing steps.

#### Molecular function

Understanding the role of mutations, in particular their contribution to diseases like cancer, requires identifying their impact on molecular functions. Causative mutations can drive cancers by activating a protein function or in-activating a function. They can promote cancer progression by their resistance to drugs or, according to a recent study, switching of functions [23].

Detection of the functional impact of mutations has not only drawn attention in cancer study, but has also been an important matter in re-sequencing efforts.

**Figure 3:**
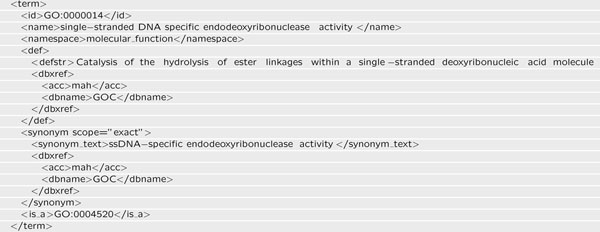
**Molecular function in OBO-XML** The example shows the molecular function *single-stranded DNA specific endodeoxyribonuclease activity* (*GO ID:0000014*), encoded in OBO-XML.

**Figure 4:**
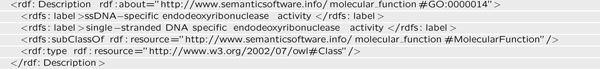
**Molecular function in RDF** For processing in OMM, the concepts are mapped from OBO-XML to RDF, here shown for the example of a molecular function, *single-stranded DNA specific endodeoxyribonuclease activity* (*GO ID:0000014*)*.*

To detect molecular functions, we use the concepts presented by the Gene Ontology. We generate an RDF representation of molecular functions from a download of the Gene Ontology. The Gene Ontology is provided in OBO-XML format, where each node is one entry (Figure 3). We first check for *molecular_function* namespaces, then, we extract the name and GO ID, as well as the synonyms of the entry. Using this information, we generate our RDF file. For obtaining further information, molecular functions are specified by their Gene Ontology ID (Figure 4). The format of a triple is C1 rdfs:subClassOf C2, where rdfs:subClassOf is an instance of rdf:Property and states that C1, here recognized as the Gene Ontology ID, is an instance of rdfs:Class and a subclass of C2, an instance of rdfs:Class, “molecular_function”. The resulting RDF is then used for gazetteering using an LKB gazetteer component [24].

#### Kinetic constants

Depending on their interests, enzyme and protein engineers apply recombinant DNA technology to improve enzyme kinetic values and stability or identify the roles of residues. Consider a study on the role of *Asn107* in humans [25]: *“To examine the role of Asn107 in the catalytic mechanism of human XR*, *mutant forms* (*N107D and N107L*) *were prepared. The two mutations increased *Km* for the substrate* (>*26-fold*) *and *Kd* for NADPH* (*95-fold*), *but only the N107L mutation significantly decreased *kcat* value.“*

Here, two prepared mutations, N107D and N107L, affect three kinetic values, Michaelis Menten constant (Km), Turn-over number (Kcat) and Dissociation constant (Kd), of the protein. To capture these kinetic properties, we manually compiled them from the scientific literature. The list of these properties is by no means exhaustive. However, property synonyms add complexity to later tasks where the relations are extracted and validated against the ontology. A simple RDF schema allows us to deal with different term representations of a concept and to resolve all aliases of the same concept. A triple is defined as C1 rdfs:subClassOf C2, where C2 is an instance of rdfs:Class, “ProteinProperty”. The rdfs:label is an instance of rdf:Property, where rdfs:domain is rdfs:resource and the rdfs:range is literal.

Normalizing all aliases to one single representation can also be helpful when populating the output ontology. Consider *Half-life* as an example, it can appear in different variations, e.g., *t0.5*, *t1/2* and *half-lives.* All these variations are represented as labels in the aforementioned RDF, thus, in case any of them matches, the mention is normalized to *Half-life.*

#### Kinetic values

Knowing the magnitude of protein properties affected by mutations enables biologists to better compare the mutation impacts of their interests. As an example, consider this bio-engineering study that was conducted on *quinoprotein Glucose Dehydrogenase* to improve the thermal stability of the enzyme [26]: *“The halflife at 55°C of Ser415Cys *(***183**** min*) *was approx ****36****-fold greater than that of the wild-type enzyme* (***5**** min*) *and ****4****-fold greater than that of the Ser231Lys variant (****40**** min*)*.”*

The *Ser* residue at position *415* is chosen for constructing different variants of the enzyme and compared with the *S231K* variant. Analyzing which variant results in the most thermostable enzyme requires the extraction of the magnitudes. Half-life of *S415C* is measured as *183 min*, whereas *S231K* was measured as *40 min*, and the measured half-lives of the two mutations are also compared to that of the wild-type enzyme.

The magnitudes of protein properties are expressed in signed numbers, decimals and ranges of values for a single parameter.

Since the existing GATE generic tokeniser [24,27] can only detect digits, we developed a simple tokeniser to capture possible representations of magnitudes. To ensure that we extract the reported ranges of magnitudes, we collected possible range representations from the literature and expressed them through grammar rules. After detecting all possible values, we check which values express a physical quantity using the patterns and discard all other values.

#### Units of measurement

Units of measurement are expressed in various formats, in mass or molar concentration (e.g., mg/ml or mmol/1), in different systems (e.g., unit, katal) and different scales (e.g., mM, *µ*M and nM). Finding how a magnitude is measured requires detecting units of measurement.

Using the same approach as for kinetic properties, the list of units of measurement was collected from the literature and encoded in an RDF schema. The RDF schema is limited to one subclass hierarchy and assigns the units of measurement to their identified concept in the OWL-DL ontology. Consider the unit of measurement, *per second*, the same concept as *PerSecond* is encoded in the OWL-DL ontology (see *Impact Ontology* Section). If any of the representations of *per second* is detected in the document, the class *PerSecond* is assigned to it, facilitating the ontology population step.

#### Physical quantities

We use the information about the units of measurement to extract physical quantities. Usually, units of measurement follow values, except for a few with no specific units of measurement, such as pH. More succinctly:

After reviewing the literature, we designed a set of patterns to capture physical quantities.

### Impact extraction component

Mutations are considered as sources of species evolution. Some result in beneficial changes, while others have detrimental effects. It is important to not only find impacts, but also to mark the origin mutation and altered protein properties for further analysis. A system capable of analyzing mutation impacts requires information from many entities. Impact analysis consists of the following steps:

1. Finding impact expressions.

2. Finding mutations or mutation keywords.

3. Identifying the polarity of the impact to detect advantageous and disadvantageous impacts.

4. Grounding the impacts to mutations to find which mutations lead to a specific impact.

5. Finding the affected protein properties.

6. Finding the magnitude of the effect to help bio-engineers compare the effects and find the most favourable mutations.

For the first step, we use ontology based gazetteering, with the help of the morphological analyzer [24], to capture term variations. Using some heuristics (see *Grounding* section), we attempt to ground the impacts to the detected mutations. Possible kinetic values are found using a custom tokeniser and validated by some rules. The magnitude of an impact is detected through heuristics and validated against the domain ontology. The last task solved by the system is to find the protein properties changed by a mutation, which is also done through additional heuristics.

#### Impact gazetteer list generation

To identify the polarity of the impacts, we use the developed OWL ontology encoding the type information of the impacts. Using an onto-gazetteer NLP component [24], the text matches the gazetteer list entries, and the impact type class in the ontology is assigned to the text. The impact gazetteer lists for *positive*, *negative*, *neutral* and *non-measurable* impacts, consisting of 130 words, were also compiled from the literature.

Furthermore, the impact terms appear in different forms. For example, *activates*, *activate*, *activated*, *activating* are all potential impact words; The problem of the term variation can be alleviated by stemming. All the aforementioned variations of *activate* have the same root: *“activate”.* The morphological analyzer [24] provides the root of the impact words, and by matching the stemming result against the prepared impact gazetteer lists, all the various representations can be detected. In the above example, by adding *activate* to our list, we can detect *activates*, *activate*, *activated*, *activating.*

#### Impact detection

Now that we gathered all the impact expressions, we will use this information to mark the impacts. The scope of the impact should be limited to the part of a sentence expressing the impact. Consider the following example [28]: *“The effects of the S136A and Y149F mutations on the Km values for NADP*(*H*) *were low*, *but the K153M mutation caused increases of more than 53-fold in the values*, *which suggests that Lys153 is involved in the coenzyme binding.”*

Three impacts are expressed in the above example:

• The effects of Y149F on the Km values for NADP(H) was low

• The effects of K158Q on the Km values for NADP(H) was low

• K153M caused increases of more than 53-fold in the values

However, this representation can not provide users with thorough information, in particular when a comparison between multiple impacts is made. Hence, we expand the scope of the impact to each sentence. In case impact words are detected in a sentence, the sentence is marked as an impact sentence. When multiple impact words are detected in a sentence, the sentence is marked multiple times as an impact sentence.

Relying on impact word expressions alone to detect impact sentences would lead to many false positives. As the next example illustrates, the impact expression ‘reduced’ exists in the sentence, however, the sentence does not express an impact of a mutation [29]: *“The limited degree of flexibility in thermophilic enzymes results in reduced catalytic efficiency when compared to that of their mesophilic counterpart at low temperatures.”*

On the other hand, extracting only the sentences containing mutation mentions and impact word expressions results in many false negatives [30]: *“Indeed*, *the ****N249Y ****substitution increases by six-fold the turnover number measured at 65C with benzyl alcohol as substrate. Furthermore*, *the affinity for coenzymes is substantially lower than that of the wt protein* (*Michaelis constant KM for p*, *25-fold greater*)*.“*

In the above example, the first impact *the increase of the turnover number* can be grounded to the mutation *N249Y.* However, the second impact on the *affinity for coenzymes* is embedded within the context. If we only extract the impact sentences containing mutations, we would ignore the second impact. Therefore, to capture impact sentences effectively, we extract all the sentences containing impact word expressions, and further filter them if no mutations or special vocabularies describing a change to a protein exist in the sentence.

The impact expressions existing in one noun phrase are considered as one expression.

Furthermore, if an impact expression appears in a verb phrase followed by another impact expression in a noun phrase, we consider them as one impact expression.

#### Impact grounding

As discussed earlier, bio-engineers are interested in knowing what kind of effects an engineered mutation can lead to. For this reason, the system must be able to accurately determine which mutation introduces a specific impact. This is accomplished by a number of heuristics.

**Figure 5:**
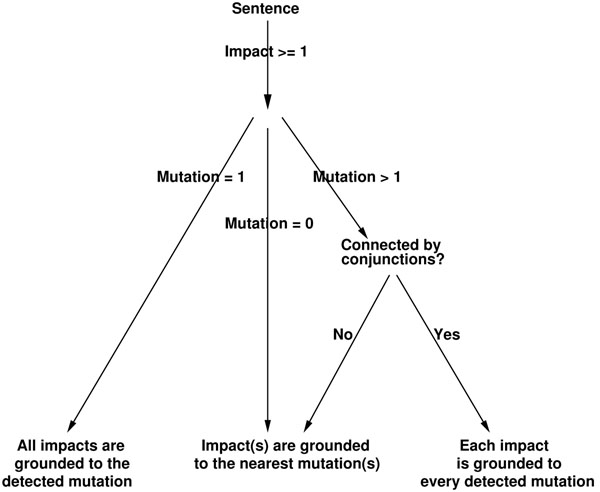
**Impact grounding heuristics** Each sentence is analysed for the occurrence of *mutation* and *impact* entities. Depending on the number of entities and the syntactic structure of the sentence, impacts are connected with mutations as shown in the figure.

Once the entities such as *mutations*, *mutation series* and *impact* words are identified and annotated, impact expressions are associated with mutations. The algorithm for semantic assignment (Figure 5) is as follows: We first check if there exist any impact expressions in a given sentence, if yes all the mutations in the sentence are collected and analysed according to the following cases.

**Case 1:** If the impact sentence contains one mutation, then all the impact expressions in the sentence are grounded to that detected mutation (the complete sentence is considered as an impact sentence). The detected mutation can be a single mutation or a mutation series.

**Case 2:** If there exists more than one mutation:

1. We check if the mutations are connected with conjunctions such as *and* and *or*; if yes, the impact is grounded to every detected mutation (the complete sentence is annotated multiple times, each time with one of the detected mutations). Some of these mutations can be mutation series, such as *N190V/W191S.*

2. Mutations or mutation series are not connected with conjunctions such as *and* and *or;* in this case, the impact is grounded to the nearest detected *mutation* or *mutations* (in case the nearest mutations are connected by conjunctions, the impact is grounded to each mutation).

**Case 3:** If no mutations are found in the sentence, the impact is grounded to the nearest mutation or mutations, making the simple assumption that the nearest mutation or mutation series invokes the impacts mentioned.

### ImpactOn relation detection

To help bio-engineers find their favourable mutation, we need to determine which protein properties are altered. Consider the following example [28]: *“In this study*, *we have confirmed the roles of Ser136*, *Tyr149 and Lys153 of XR as the catalytic triad by drastic loss of *activity* resulting from the mutagenesis of S136A*, *Y149F and K153M in rat XR.”*

Two prerequisite pieces of information, the impact expression, *loss* and the protein property changed by the mutation, *activity* are detected. Now we attempt to associate the appropriate pair. We use a simple heuristic to detect which protein property is affected by a mutation:

1. We first check if there exists one impact in a given sentence, if so the sentence is searched for protein properties. We assume that all detected protein properties are altered by the impact.

2. If multiple impacts are detected in a sentence, each impact is linked to the nearest protein property.

The *impactOn* relation is represented with the sentence containing the impact expression and the protein property. The result annotation of the above example is shown in Table 4.

**Table 4:** Result annotation example

impactOn	… of Ser136, Tyr149 and Lys153 of XR as the catalytic triad by drastic loss of activity …
**Protein property**	activity

**Impact Expression**	loss

The ontology property ‘impactOn’ connects impact expressions with the affected protein property.

### MeasuredWith relation detection

At this stage, we find relations between the protein property affected by an impact and units of measurement and effect magnitudes (numerical values). Consider the following two examples: (1) [29]* “The mutant SsADH displays improved *thermal stability, *as indicated by the increase in Tm from ****90 to 93°C***, *which was determined by the apparent transition curves.”* (2) [31]* “Except for Thr416Val/Thr417Val*, *which had a *Km* value of ****16 mM***, *the mutants had *Km* values identical to ****20 mM*** Km* value of the wild-type enzyme.“*

In the first example, to know how much the *thermal stability* was improved by the mutation, we need to link the extracted protein property, *thermal stability*, with the physical quantity, *90 to 93°C.* In the second example, we need to detect that the *Km* property of the protein affected by the double mutant, *T416V/T417V*, is measured as *16 mM*, while other mutants had the same *Km* value as that of the wild-type enzyme, which is measured as *20 mM.* To fulfil our objective of relating the protein properties with their units of measurement, we use simple proximity heuristics. The detected relation candidates are then validated against the domain ontology (see *Impact Ontology* section): If the detected physical quantity is not among the possible value fillers of the slots for the aforementioned protein property, the relation candidate is discarded. Consider the following example [32]: *“In addition*, *the* half lives *at ****60°C**** of the R156E and N173D xylanases were respectively 6 and 40 min longer than that of the wild-type enzyme even in the absence of substrate.”*

The protein property *half lives* is measured with *minute*, *hour* and *fold;* in the above example, the closest physical quantity is *60°C*, and once the relation is validated against the ontology, it is discarded as *Degree Celsius* is not one of the fillers of *half life* and the correct filler *6 and 40 min* is assigned.

To represent the relation, we mark the sentence as a *measuredWith* relation with two features, property name and physical quantity.

### Ontology population

To provide protein engineers and scientists with comprehensive information and a more expressive model, we populate our domain ontology with the extracted information, which can then be queried as a knowledge base. Since manually populating the ontology is a cumbersome task, we integrated the OwlExporter [33,34] component to automate this task.

Two ontologies are required to export our extracted information, our domain Impact ontology and a NLP ontology provided with the OwlExporter component [34]. The NLP ontology contains concepts such as Document and Sentence.

While populating our domain ontology, the OwlExporter automatically populates the NLP ontology. Individuals of our domain concepts, such as Mutation, Mutationlmpact and ProteinProperty are associated with the individuals of the NLP concepts, such as Sentence. We can then invoke more advanced queries, e.g., finding all the extracted impacts in a specific sentence.

In order to be able to export the entities and the relationships to our domain ontology, we need to assign OWLExportClass and OWLExportRelation annotation types to the document annotations. This is achieved with additional JAPE grammar rules. By assigning these two types of annotations to our document annotations, we inform the OwlExporter about the annotations we want to export.

**Figure 6:**
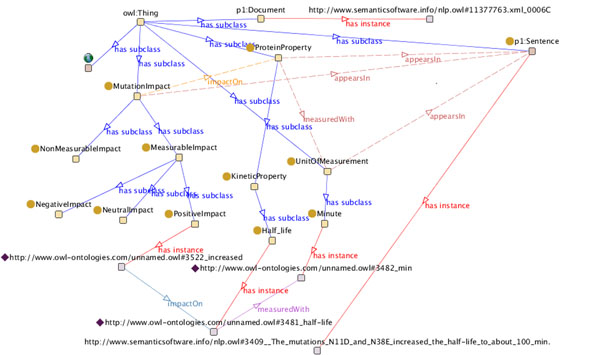
**An example of the populated impact ontology** OMM can export results into the ontology shown in Figure 2 through so-called *ontology population.* Each occurrence of a mutation, impact, etc. is connected with the corresponding domain concepts and additionally linked to its source *sentence* and *document.*

Figure 6 shows an example from the populated ontology with sentence and impact instances.

### Application

Our system is implemented based on the *General Architecture for Text Engineering* (GATE) [24], a Java-based open source component framework for text processing. Our system can be run stand-alone, embedded in other applications, or deployed on a cloud computing infrastructure for large-scale document processing using the GATE Cloud Parallelizer (GCP). Additionally, we provide a number of semantic access methods, described below.

#### Web Service invocation

**Figure 7:**
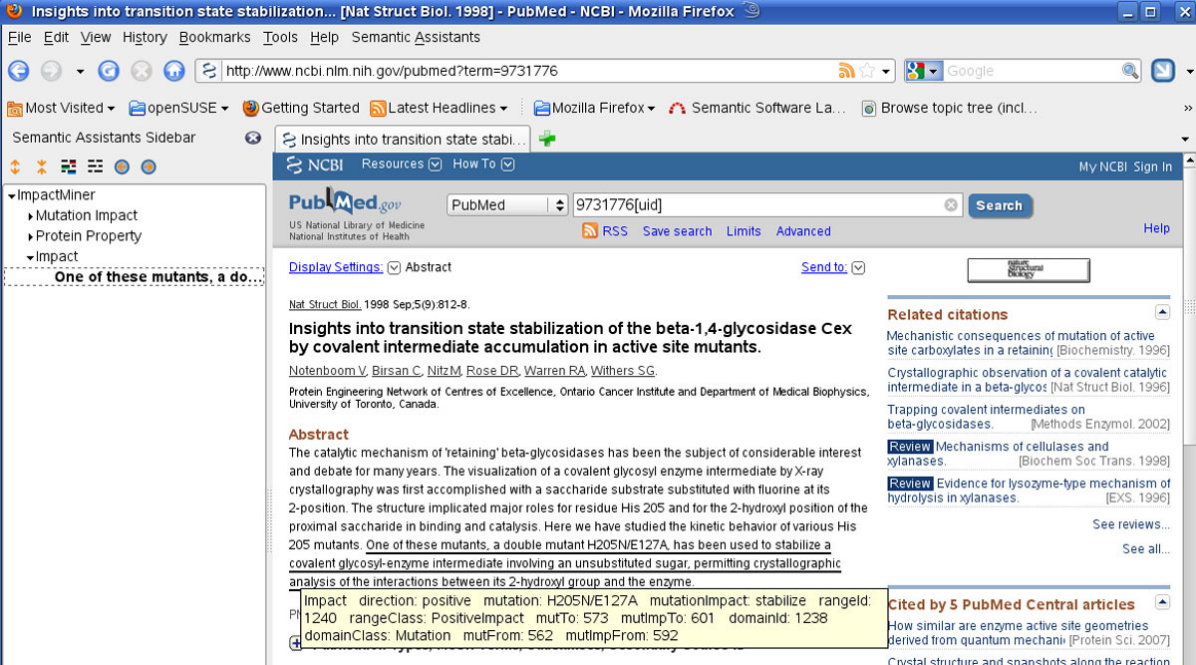
**OMM web browser integration** Impact extraction can be dynamically requested from the Firefox browser, which is then executed through the OMM Semantic Assistants web service. Results are mapped as annotations onto the viewed document, which facilitates scientific literature analysis.

To use our pipelines as a web service, we created OWL service descriptions for the Semantic Assistants framework [35]. Two services are currently provided, one for mutation tagging and one for impact detection. These services are described through metadata expressed in an OWL ontology. Both services can then be deployed in a Semantic Assistants server. The server allows any web client to send documents to the service through standard web service invocations and receive the results in XML format. Additionally, Semantic Assistants-enabled clients, like OpenOffice or the Firefox web browser (Figure 7), can directly send documents to the services on behalf of a user.

#### Querying impact information

**Figure 8:**
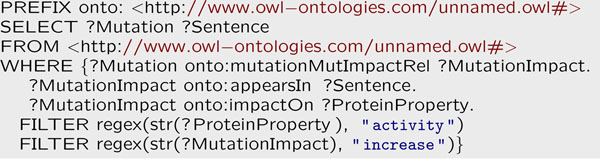
**SPARQL query example** Text mining results can be obtained by querying the populated ontology. The example shown here is the SPARQL translation of the question, *find all mutations that increased the activity of a protein.*

**Figure 9:**
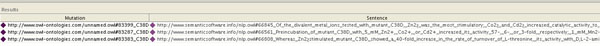
**Query Result** Example output from the query shown in Figure 8, listing all mutations that *increased* the protein property “activity”.

Presenting impact information in a structured format allows users to quickly access the relevant information [36]. For example, an end user might be interested to search for impacts of a specific mutation, or all the altered properties of an impact. Towards this end, we export the extracted information to the ontology; consequently, we can simply query the ontological knowledge base for our desired information. Figure 8 simply queries for the mutations that increased the activity of a protein using the SPARQL query language; The results of this query are shown in Figure 9.

## Results

We analyzed the performance of our approaches for mutation series and impact extraction in detail on different corpora. First, the evaluation of the mutation series detection module is investigated. Then, the effectiveness of the impact extraction, as well as grounding to the correct mutation is measured on literature describing enzymes.

### Data

To evaluate the performance of the system for each task, we prepared two corpora: *Mutation Series* and *Impact.*

#### Mutation series detection corpus

We prepared a corpus containing 11 full-text PubMed articles on enzymes to assess the efficiency of the system in detecting mutation series. We ensured that all these documents contain multiple mutation mentions. These documents contain a total of 1306 mutations and 271 mutation series. The list of documents used for evaluation is provided in an additional file [see Additional file 1].

#### Impact extraction corpora

We selected 40 PubMed IDs and manually annotated them with the impact information. For each impact mention, only the part of the sentence mentioning the mutation and the impact was selected. Thus, if a sentence expresses multiple impacts, all are annotated separately [see Additional file 2 for manual annotations]. The impacts are grounded to the respective mutations and the EC number of experimented enzymes is specified. The list of documents used for evaluation is provided in an additional file [see Additional file 1].

### Evaluation

First, the correctness of the mutation series extraction is assessed. Second, the effectiveness of the impact extraction, as well as grounding to the correct mutation, is measured on literature describing enzymes. Since the mutation series detection relies on correctly recognizing mutations, we first show the mutation detection result for each system, followed by the result of our mutation series detection.

#### Quantitative evaluation metrics

The evaluation procedure is performed by comparing the manually annotated texts with the annotations generated by our system, measured with the metrics explained in this section. The number of correctly identified items as a percentage of the number of items identified is specified as *precision* (P). The number of correctly identified items as a percentage of the total number of correct items is defined as *recall* (R). The *F-measure* (F) is used as a weighted (geometric) average of precision and recall. Finally, *accuracy* is the percentage of decisions that are correct. The performance results are computed according to different criteria: *Strict* (S) and *Lenient* (L). In “Strict”, we measure all partially correct responses as incorrect. In “Lenient”, all partially correct responses are measured as correct [24].

#### Mutation detection evaluation

Both MutationFinder [6] and our OMM MutationTagger were applied to 11 manually annotated documents; the comparative results of the systems are shown in Table 5.

**Table 5:** Mutation detection evaluation

Mutation detection using MutationFinder										
**Document (PMID)**	**Correct**	**Partially C.**	**Missing**	**Spurious**	**Strict**			**Lenient**		
					
					**P**	**R**	**F**	**P**	**R**	**F**

10860737	200	0	0	2	99%	100%	100%	99%	100%	100%
12604240	90	0	0	1	99%	100%	99%	99%	100%	99%
12664592	24	0	0	0	100%	100%	100%	100%	100%	100%
12702265	46	0	11	14	77%	81%	79%	77%	81%	79%
12890481	92	0	0	0	100%	100%	100%	100%	100%	100%
12902331	173	0	7	5	97%	96%	97%	97%	96%	97%
15026177	142	0	5	0	100%	97%	98%	100%	97%	98%
17761677	49	0	0	0	100%	100%	100%	100%	100%	100%
19143837	154	0	3	0	100%	98%	99%	100%	98%	99%
9731776	2	0	0	0	100%	100%	100%	100%	100%	100%
14592457	170	0	138	3	98%	55%	71%	98%	55%	71%

**Average**	1142	0	164	25	98%	87%	92%	98%	87%	92%

**Mutation detection using MutationTagger**										

**Document (PMID)**	**Correct**	**Partially C.**	**Missing**	**Spurious**	**Strict**			**Lenient**		
					
					**P**	**R**	**F**	**P**	**R**	**F**

10860737	191	0	9	5	97%	96%	96%	97%	96%	96%
12604240	89	0	1	1	99%	99%	99%	99%	99%	99%
12664592	24	0	0	0	100%	100%	100%	100%	100%	100%
12702265	41	0	16	0	100%	72%	84%	100%	72%	84%
12890481	91	0	1	0	100%	99%	99%	100%	99%	99%
12902331	171	1	9	4	98%	95%	96%	98%	95%	96%
15026177	146	0	1	1	99%	99%	99%	99%	99%	99%
17761677	49	0	0	0	100%	100%	100%	100%	100%	100%
19143837	148	0	9	0	100%	94%	97%	100%	94%	97%
9731776	2	0	0	0	100%	100%	100%	100%	100%	100%
14592457	297	0	11	0	100%	96%	98%	100%	96%	98%

**Average**	1249	0	57	11	99%	96%	97%	99%	96%	97%

Comparative evaluation of the mutation detection performance, using MutationFinder and OMM MutationTagger.

#### Mutation series evaluation

We also verified the correctness of the extracted mutation series. The results are presented in Table 6.

**Table 6:** Mutation series detection evaluation

Mutation series detection (MutationFinder)										
**Document (PMID)**	**Correct**	**Partially C.**	**Missing**	**Spurious**	**Strict**			**Lenient**		
					
					**P**	**R**	**F**	**P**	**R**	**F**

10860737	13	0	0	0	100%	100%	100%	100%	100%	100%
12604240	11	0	0	0	100%	100%	100%	100%	100%	100%
12664592	4	0	0	0	100%	100%	100%	100%	100%	100%
12702265	1	0	7	0	100%	12%	22%	100%	12%	22%
12890481	26	0	0	0	100%	100%	100%	100%	100%	100%
12902331	51	0	0	0	100%	100%	100%	100%	100%	100%
15026177	13	0	0	0	100%	100%	100%	100%	100%	100%
17761677	1	0	0	0	100%	100%	100%	100%	100%	100%
19143837	40	0	0	0	100%	100%	100%	100%	100%	100%
9731776	1	0	0	0	100%	100%	100%	100%	100%	100%
14592457	1	0	102	0	100%	1%	2%	100%	1%	2%

**Average**	162	0	109	0	100%	60%	75%	100%	60%	75%

**Mutation series detection (MutationTagger)**										

**Document (PMID)**	**Correct**	**Partially C.**	**Missing**	**Spurious**	**Strict**			**Lenient**		
					
					**P**	**R**	**F**	**P**	**R**	**F**

10860737	13	0	0	0	100%	100%	100%	100%	100%	100%
12604240	11	0	0	0	100%	100%	100%	100%	100%	100%
12664592	4	0	0	0	100%	100%	100%	100%	100%	100%
12702265	7	0	1	0	100%	88%	93%	100%	88%	93%
12890481	26	0	0	0	100%	100%	100%	100%	100%	100%
12902331	51	0	0	0	100%	100%	100%	100%	100%	100%
15026177	13	0	0	0	100%	100%	100%	100%	100%	100%
17761677	1	0	0	0	100%	100%	100%	100%	100%	100%
19143837	40	0	0	0	100%	100%	100%	100%	100%	100%
9731776	1	0	0	0	100%	100%	100%	100%	100%	100%
14592457	102	0	1	0	100%	99%	100%	100%	99%	100%

**Average**	269	0	2	0	100%	99%	100%	100%	99%	100%

#### Impact analysis evaluation

Here, we analyse how correctly our system can detect all impacts expressed in a sentence. We further investigate the performance of our developed grounding algorithm (see *Grounding* Section). The performance of our system on our manually annotated corpus of 40 documents is assessed and the results are summarized in Tables 7 and 8. OMM system results are provided in an additional file [see Additional file 3].

**Table 7:** Impact Detection Evaluation on 40 full-text Documents

Impact Detection Evaluation									
	**MutationTagger**			**MutationFinder**			**MutationTagger+ MutationFinder**		

**#Documents**	**Precision**	**Recall**	**F-Measure**	**Precision**	**Recall**	**F-Measure**	**Precision**	**Recall**	**F-Measure**

40	70.4%	71.3%	70.8%	71.1%	71.4%	71.24%	70.8%	71.5 %	71.1%

**Table 8:** Impact Grounding Evaluation on 40 manually annotated documents

	Impact Grounding Evaluation – MutationTagger
**Accuracy**	76.5%

	**Impact Grounding Evaluation – MutationFinder**

**Accuracy**	76.9%

	**Impact Grounding Evaluation – MutationTagger + MutationFinder**

**Accuracy**	77%

In our corpora, 5% of all point mutations are expressed in natural language; thus, in an experiment, we considered the results of both MutationTagger and MutationFinder for the impact detection and grounding tasks. As can be seen in Table 7, this combination of both systems slightly increases recall at the expense of precision.

## Discussion

False negatives of impact detection are mainly due to author-defined mutation names. For example, PMID 10074357, reporting on the mutant of *alcohol dehydrogenase*, uses *mSsADH* to refer to *N249Y* in the document. Authors of the paper PMID 10544015 also assign *No. 87* to a mutation containing 8 amino acid substitutions; *T71A*, *K264E*, *L317S*, *T331A*, *R407L*, *S415G*, *K455I and E277G.* Since we rely on mutation mentions and the keywords introduced earlier in *Impact Detection* section to detect impacts, these impacts are not detected.

Tables from processed PDF files are converted into indistinct textual blocks, and in case they are reporting the impacts of mutations, our system detects them as impacts. These mentions are not manually annotated, thus they are considered as false positives.

## Conclusions

Mutation impacts are essential for understanding the role of mutations. The data regarding the mutations and impacts exists primarily in scientific publications. In this paper, we described Open Mutation Miner (OMM), a comprehensive, modular, open source text mining system for extracting and grounding mutation impacts, affected protein properties and magnitudes of effects.

The performance of our system is evaluated on multiple corpora. Furthermore, we created additional manual annotations for the biomedical literature. Our ontology population approach provides comprehensive information to a biologist and can be queried or further integrated with other systems.

Further work will address mutation co-reference resolution; In journal papers, very often the authors use pronominal or nominal mutation references that hinders the grounding of impacts. All occurrences of mutations, including nominal and pronominal references are required to be detected. Deletion and insertion mutations pose additional challenges to be addressed in a future version.

## Abbreviations used

AMENDA: Automatic Mining of ENzyme DAta; BRENDA: BRaunschweig ENzyme DAtabase; DL: Description Logic; EC: Enzyme Commission; FRENDA: Full Reference ENzyme DAta; GATE: General Architecture for Text Engineering; GO: Gene Ontology; JAPE: Java Annotation Pattern Language; KID: KInetic Database; NLP: Natural Language Processing; OWL: Web Ontology Language; RDF: Resource Description Framework; SI: Systéme International d’unités; SPARQL: SPARQL Protocol and RDF Query Language; XML: eXtensible Markup Language.

## Authors’ contributions

NN designed the impact extraction and grounding rules, implemented the system, contributed to the ontology design, annotated the documents, and performed the evaluation. RW provided GATE and NLP expertise, co-ordinated the overall project, contributed to the ontology and the Semantic Assistants framework. Both authors contributed to the manuscript.

## Competing interests

The authors declare that they have no competing interests.

## Supplementary Material

Additional file 1 **List of documents used for evaluation** The list of documents (PubMed IDs) used in the corpora for mutation series and impact analysis evaluation. Click here for file

Additional file 2 **Manual annotations** Manual annotations of 40 documents used for evaluation, presented in tab-delimited format. Click here for file

Additional file 3 **OMM system results** The output of our OMM system, listing all detected impacts that are grounded to their respective mutations. Click here for file
