# Supplementary material for: Automated extraction and semantic analysis of mutation impacts from the biomedical literature
Source: BMC Genomics. 2012 Jun 18;13(Suppl 4):S10. doi: 10.1186/1471-2164-13-S4-S10 (PMC3395893; doi:10.1186/1471-2164-13-S4-S10)
Supplement: Additional file 1 — List of documents used for evaluation The list of documents (PubMed IDs) used in the corpora for mutation series and impact analysis evaluation. [file 1471-2164-13-S4-S10-S1.pdf]

1 Mutation Series Corpora

The list of documents (PubMed IDs) prepared for mutation series evaluation is presented in Table 1.

Table 1: List of documents (PubMed IDs) prepared for mutation series evaluation

10860737	12604240	12664592	12702265	12890481
12902331	14592457	15026177	17761677	19143837
9731776				

2 Impact Corpora

The lists of documents used for impact analysis module are listed in this section.

Table 2: List of documents (PubMed IDs) manually annotated for impact evaluation

10074357	10544015	10955993	11265460	12205101
12604240	12650918	12702265	12746550	12890481
12902331	14996818	15026177	15103634	15178335
15206895	15276835	15311930	15625320	15882618
16000301	16129418	16139296	16242114	17581819
17952367	17969139	17974571	19398559	19653994
19674460	8706817	9705344	9784233	16889958
19290871	17914867	18571493	17343568	17420465
